# Dynamic Transcriptomic and Phosphoproteomic Analysis During Cell Wall Stress in *Aspergillus nidulans*

**DOI:** 10.1074/mcp.RA119.001769

**Published:** 2020-11-23

**Authors:** Cynthia Chelius, Walker Huso, Samantha Reese, Alexander Doan, Stephen Lincoln, Kelsi Lawson, Bao Tran, Raj Purohit, Trevor Glaros, Ranjan Srivastava, Steven D. Harris, Mark R. Marten

**Affiliations:** 1 Department of Chemical, Biochemical, and Environmental Engineering, University of Maryland Baltimore County, Baltimore, Maryland, USA; 2 Center for Plant Science Innovation and Department of Plant Pathology, University of Nebraska-Lincoln, Lincoln, Nebraska, USA; 3 Department of Chemical and Biomolecular Engineering, University of Connecticut, Storrs, Connecticut, USA; 4 BioScience Mass Spectrometry Facility, The U.S. Army CCDC Chemical Biological Center, BioSciences Division, Aberdeen Proving Ground, Maryland, USA; 5 Department of Biological Sciences, University of Manitoba, Winnipeg, Manitoba, Canada; 6 BioSciences Division, B11 Bioenergy and Biome Sciences, Los Alamos National Laboratory, Los Alamos, New Mexico, USA

**Keywords:** Signaling molecules, phosphoproteome, gene expression, pathway analysis, molecular dynamics, cell wall signaling, cell wall strength, filamentous fungi, transcriptomics

## Abstract

The fungal cell-wall integrity signaling (CWIS) pathway regulates cellular response to environmental stress to enable wall repair and resumption of normal growth. This complex, interconnected, pathway has been only partially characterized in filamentous fungi. To better understand the dynamic cellular response to wall perturbation, a β-glucan synthase inhibitor (micafungin) was added to a growing *A. nidulans* shake-flask culture. From this flask, transcriptomic and phosphoproteomic data were acquired over 10 and 120 min, respectively. To differentiate statistically-significant dynamic behavior from noise, a multivariate adaptive regression splines (MARS) model was applied to both data sets. Over 1800 genes were dynamically expressed and over 700 phosphorylation sites had changing phosphorylation levels upon micafungin exposure. Twelve kinases had altered phosphorylation and phenotypic profiling of all non-essential kinase deletion mutants revealed putative connections between PrkA, Hk-8–4, and Stk19 and the CWIS pathway. Our collective data implicate actin regulation, endocytosis, and septum formation as critical cellular processes responding to activation of the CWIS pathway, and connections between CWIS and calcium, HOG, and SIN signaling pathways.

## Teaser

The transcriptome and phosphoproteome of the filamentous fungus *Aspergillus nidulans* was monitored after cell wall stress. Over 1800 genes and 700 phosphorylation sites showed dynamic expression and occupancy, respectively. These data guided selection of kinase deletion strains for testing for cell-wall related phenotypes. Kinase-substrate analysis revealed possible connections between CWIS, SIN, HOG, and calcium pathways. Fungi exposed to wall stress experience an increase in septation formation, which may function as a biological defense mechanism.

## Graphical Abstract

**Figure:**
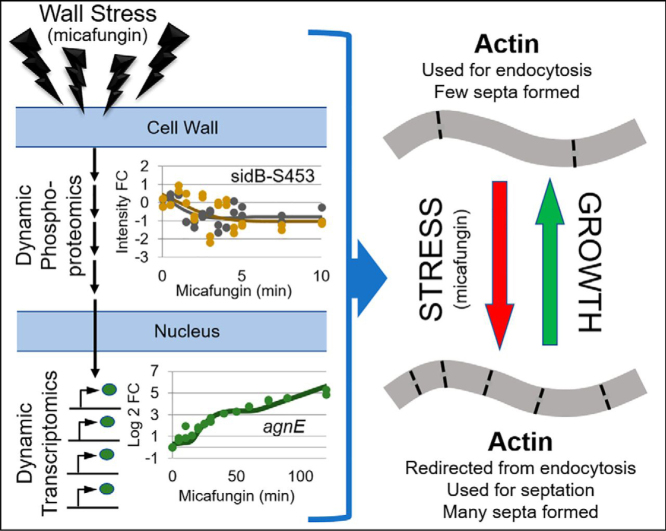

**Highlights**

• Dynamic transcriptomic and phosphoproteomic study of response to cell-wall stress.
• MARS model identifies significantly dynamic gene expression and phosphorylation.
• Septation formation increases during cell-wall stress (via micafungin exposure).
• Cell wall phenotype assays identified 8 putative CWIS related kinases.

The cellular signaling network is a complex system that controls nearly all cellular processes underlying growth, metabolism, morphogenesis, and development (1, 2). Generally, cell surface receptors are perturbed by environmental stimuli and transduce information to the nucleus via signaling proteins, which typically include G-proteins, kinases, and transcription factors. A signaling pathway of interest in fungi is the cell wall integrity signaling (CWIS) pathway. The CWIS is nominally responsible for wall maintenance and repair (3), which are critical as fungi rely on the wall for protection, shape, strength, and host invasion (4, 5, 6, 7). The CWIS is also of interest as cell walls are present in fungi, but absent in humans, thereby making the fungal cell wall an ideal target for antifungal therapeutics (8, 9, 10).

The CWIS is activated in response to wall stress and/or damage (3) and is composed of a conserved set of proteins across a variety of fungal species including *Aspergillus nidulans* (11), *A. fumigatus* (12), *A. oryzae* (13), *Saccharomyces cerevisiae* (3), *Schizosaccharomyces pombe* (14), and *Candida albicans* (15). In the model fungus *A. nidulans*, the CWIS pathway is composed of cell surface receptors which sense cell wall stress (16). These sensors activate the guanine nucleotide exchange factor (GEF), Rom2, which acts on the GTPase RhoA to trigger activation of PkcA (16, 17, 18, 19). This subsequently activates a MAPK cascade, which includes BckA-MkkA-MpkA (11, 20, 21). MpkA, the final kinase in this cascade, activates the transcription factor RlmA which is responsible for controlling expression of α-1,3-glucan synthase genes (11).

Notably, unlike *S. cerevisiae*, the transcription of many cell wall related genes (including β-1–3-glucan and chitin synthase genes) in *A. nidulans* is regulated in an MpkA-independent manner (11). In an effort to characterize the CWIS pathway, several studies have sought out this alternative signaling pathway to identify how β-1,3-glucan and chitin synthase genes are regulated in response to cell wall perturbation (22, 23, 24, 25, 26, 27). Connections between the CWIS pathway and several other pathways have also been identified. Examples include the unfolded protein response (UPR) pathway, calcium signaling, branching regulation, iron homeostasis, the high osmolarity-glycerol (HOG) pathway, and the cyclic AMP protein kinase A (PKA) pathway (17, 28, 29, 30). These pathway networks are complex and highly interconnected, making it difficult to resolve network topography. We are interested in developing a better understanding how cell wall integrity signaling is transduced to the nucleus and identifying downstream effectors whose expression is controlled by this process.

The most common post translational modification (PTM) mediating signal transduction is phosphorylation (1). To capture the global phosphorylation-state of the cell, mass spectrometry (MS) has emerged as a powerful tool. In the past decade, major technological advances in MS have enabled researchers to accurately identify and quantify thousands of phosphorylation sites from a single run (31). However, although a single MS run provides an abundance of information, when studying signaling behavior it is important to consider the dynamics of protein phosphorylation. Phosphorylation events typically occur rapidly (within minutes), thus to capture signaling progression, multiple samples must be taken over a short time span (32). In addition, evaluating dynamic changes in phosphorylation can aid in determining which events are more likely to be direct interactions of kinases and substrates (33).

In this work, we study the dynamic response of the cell wall integrity signaling pathway to a wall perturbation. Micafungin, a β-glucan synthase inhibitor, is used as a cell wall perturbant as it is known to directly activate the CWIS pathway (11). We used quantitative, label-free, mass spectrometry (over a short period of time; 10 min) to assess dynamic changes in protein phosphorylation and transcriptomic analysis (over a longer period of time; 120 min) to assess changes in global gene-expression levels. Overall, we identified over 700 dynamic phosphorylation sites, which includes 15 sites on kinases and 16 on putative transcription factors. Over 1800 significantly dynamic genes were expressed upon micafungin treatment, 25 of which have previously been identified as cell-wall related. Our findings reveal that the coordinated response of the calcium, HOG, and Septation Initiation Network (SIN) pathways are involved in the response to cell wall stress. Furthermore, our results underscore the potential of this multi-omics approach to study signaling networks in general.

## EXPERIMENTAL PROCEDURES

##### Strains and Media

*Aspergillus nidulans* A1405 (Fungal Genetics Stock Center; FGSC) was used as the control strain. Frozen stocks were spread on MAGV plates (2% malt extract, 1.5% agar, 2% glucose, 2% peptone, and 1 ml/L Hutner's trace elements and vitamin solution) and incubated for 2 days at 28 °C (29). 1E7 spores were harvested and inoculated into 50 ml of YGV (pH 3.3) (0.5% yeast extract, 1% glucose, and 1 ml/L Hutner's trace elements and vitamin solution). Culture was grown in a 250 ml baffled flask at 250rpm and 28 °C. After 12 h growth, this flask was used to seed 1.2L YGV in a 2.8L Fernbach flask. Deletion strains used in this study are all available from FGSC. Deletion strains are: Δ*mpkA* (A1404), Δ*stk19* (A1350), Δ*prkA* (A1299), Δ*yak1* (A1351), Δ*hk-8–4* (A1389), Δ*chkC* (A1369), Δ*schA* (A1332), Δ*cmkC* (A1391), Δ*npkA* (A1354), Δ*nrc2* (A1284), Δ*srrB* (A1370), Δ*kin1* (A1307).

##### Western Blotting

After ∼20 h of growth (mid-exponential growth phase), 20 ng/ml micafungin per 1g/kg dry cell weight (DCW) was added to the control strain culture. DCW was determined by removing ∼25 ml of culture from the shake flask during growth. Amount of culture removed was determined by weight (*i.e.* “wet weight” kg culture). Fungal biomass was recovered from the liquid via vacuum filtration, and then dried at 100 °C before determining “dry weight.” Dry cell weight (DCW) is the concentration of biomass in culture, and was calculated as “dry weight” divided by the “wet weight.” Twenty-five milliliters of culture was removed after 0, 1, 3, 5, 10, 15, and 30 min of micafungin exposure and mycelia were separated from broth and immediately frozen in liquid nitrogen. Frozen mycelia were crushed with mortar and pestle into a fine powder and mixed with TNE buffer at 1:1 (v/v) (20 mm Tris-HCl pH 7.4, 150 mm NaCl, 2 mm EDTA). Samples were spun at 10rpm at 4 °C for 10 min then centrifuged at 4 °C and 10,000xG for 10 min and BCA assay (Pierce, Rockford, IL) was performed on the supernatant. One hundred micrograms of protein was exposed to SDS-page electrophoresis and transferred to a nitrocellulose membrane (Life technologies, Carlsbad, CA). Western blotting preparation steps were performed as described in Chelius *et al.*, 2019. Two biological replicates were modeled using Michaelis-Menten kinetics.

##### Dynamic Micafungin Treatment, Experimental Design, and Phosphoproteomic Sample Preparation

Fungi were grown until mid-exponential phase (about 20 h) and 20 ng micafungin/ml culture per 1g/kg DCW was added. About 25 ml of culture was removed from the flask while the shaker was still shaking (ensuring a homogeneous sample) at 0, 30s, 1, 1.5, 2, 2.5, 3, 3.5, 4, 4.5, 5, 7.5, and 10 min after micafungin exposure. Immediately after removing sample, the fungal mass separated from broth, frozen in liquid nitrogen, and stored at −80 °C. This experiment was run with two biological replicates.

Samples harvested from shake flask culture were prepared for phosphoproteomic analysis following methods of Chelius *et al.*, 2019. Briefly, frozen biomass was crushed by mortar and pestle with liquid nitrogen, mixed with 1:1 v/v TNE buffer, and the BCA assay (Pierce, Rockford, IL) was used to determine protein concentration. The Filter aided sample preparation (FASP) was used to digest protein (29) and then 500 μg protein was incubated with Trypsin Gold (Promega, Madison, WI) (50 μg protein: 1 μg trypsin) overnight in a 37 °C water bath. The samples were lyophilized to dryness and phosphopeptides were collected using the Pierce High-Select TiO_2_ Phosphopeptide Enrichment Kit (Pierce, Rockford, IL) protocol. Samples were dried to completeness and stored at −80 °C until mass spectrometry analysis.

##### LC-MS/MS and LFQ Analysis

Stored samples were reconstituted in 20 μl of acetonitrile/water/formic acid 5/95/0.5 (v/v/v) and run in duplicate on a Dionex Ultimate 3000 nanoLC system coupled to an Orbitrap Fusion Tribrid mass spectrometer (Thermo Scientific, San Jose, CA). Separation of peptides was performed on EASY spray C18 75 μm × 50 cm column for a 190 min gradient at flow rate of 200 nL/min using mobile phase A of 0.1% formic acid in water and mobile phase B of acetonitrile/water/formic acid 80/20/0.1 (v/v/v). Mass spectrometry data were collected in positive ionization mode using a data dependent acquisition method with a full MS scan for *m*/*z* range 350–1500 in orbitrap at 120 K resolution. Consecutive MS/MS scans were performed in the ion trap by top-speed decision selection with a dynamic exclusion of 20 s. Precursor ions selected from the first MS scan were isolated with an isolation width of 1.6 *m*/*z* for collision induced dissociation (CID) energy and normalized collision energy (NCE) set to 30.

Samples from both biological replicates and two technical replicates for all timepoints (52 samples) were processed simultaneously with MaxQuant software version 1.5.3.17 (34). Database search was completed using Andromeda search engine (which is a part of MaxQuant) after quantification. MaxQuant analysis was performed as described in Chelius *et al.*, 2019 to quantify peptide abundance. MaxQuant automatically quantified phospho-peptides using relative quantification of ion intensities which compares the abundance of the same peptide species across all runs. Parameters that were used include: fixed modification of carbamidomethyl (C), variable modifications of phosphorylation (STY), acetylation (N terminus), and oxidation (M), trypsin as the protease, and 2 missed cleavages. The mass tolerance for precursor ions was set 20 ppm in the first search and 4.5 ppm in the main search, and the fragment mass tolerance was set to 20 ppm. The FDR was set to 0.01. MS/MS spectra were searched against a target database consisting of 10,555 entries from the UniprotKB database (UP000000560). Combining a high number of timepoints (13) with several replicates (2 biological replicates with 2 technical replicates) ensures a sample size large enough to provide statistical power. Peak lists from all MS/MS spectra were extracted from MaxQuant result files using MS-viewer (35) and can be accessed at http://msviewer.ucsf.edu/prospector/cgi-bin/msform.cgi?form=msviewer (search key: yhspxctyvc).

##### Experimental Design and Statistical Rationale

Phosphoproteomic analysis was completed on two biological replicates each containing 13 timepoints. All samples were run in duplicate for a total of 52 mass spectrometry runs. The control is the first time point (0 min) which was taken immediately before micafungin addition (*i.e.* cell perturbation). To determine if the phosphorylation level is significantly dynamic, multivariate adaptive regression splines (MARS) model (36) was used following a method developed by Oliveira *et al.*, 2018. This method ensures that our dynamic response is not background noise.

##### Phospho-site Filtering and Statistical Assessment

Perseus 1.6.1.1 software was used to filter phosphorylation sites (37). Sites were removed if there was a contaminant, reverse match, or the location probability was less than 0.75. Next, we applied filtering rules to the biological replicates separately. A phosphorylation must be present in both technical replicates of at least 10 of the 13 time points and present in either the initial time point (0 min) or the 30 s time point (our control) (33). Finally, these rules applied to both biological replicates resulted in a total of 925 highly confident phosphorylation sites. For the remainder of the analysis the intensity values were transformed into log 2-fold changes. A MARS model was fit to the fold changes from 26 (13 time points, 2 technical replicates) samples using ARESLab, a Matlab package (38). The arespredict function (with default settings) determined the quality of fit (mean square error; MSE) of the actual data and 10,000 random permutations of the fold change levels. The *p* value was calculated as the fraction of randomly permutated models with better fit than the actual model. If the *p* value was less than 0.05 the phosphorylation sight was deemed significantly responsive (Class I sites are significant in both biological replicates, Class II are significant in 1 biological replicate).

##### RNA Sequencing and Statistical Analysis

Fungi were grown and treated with micafungin identically to phosphoproteomic samples. Samples were taken at 0, 5, 10, 15, 20, 25, 30, 40, 50, 60, 75, 90, and 120 min for three biological replicates. Harvested mycelia were frozen with liquid nitrogen and ground into a fine powder by mortar and pestle. RNA extraction and preparation was completed as previously described (39). The samples were sent to the University of Nebraska Medical Center's Bioinformatics and Systems Biology Core for library preparation and RNA Sequencing (https://unmc.edu/bsbc). The raw samples were of single end reads of high quality in 4 separate lanes in FASTQ files. The following costume RNA-Sequencing pipeline was used: HISAT2 2.1.0 (40), HTSeq-Count 0.9.1(41), DESeq2 1.20.0 (42). The annotation file (Gene Transfer File) and whole genome of wildtype strain FGSC A4 FNA file was supplemented from the database Ensemble Fungi (43).

Like the phosphoproteomic data, genes were required to have reads in 2 out of 3 replicates in all 13 timepoints. The normalized reads were transformed into log 2-fold change values. Finally, the dynamic test (MARS model) described above was applied to all replicates. Significance threshold was set to *p* < 0.01.

##### Predictive Software - PHOSIDA, NetworKIN, STRING, GO and Cluster Analysis

Several software tools were used to further analyze the phosphorylation hits. PHOSIDA analyses all phosphorylation motifs to identify statistically overrepresented motifs (http://141.61.102.18/phosida/index.aspx) (44, 45). PHOSIDA parameters were set to the following: minimum score at 10 and minimum proportion of matching sites was 5% (46). Dynamically phosphorylated sites were analyzed by NetworKIN to predict which kinases target specific phosphorylation sites from the yeast proteome (http://networkin.info/) and phosphosite-kinase interactions with a score greater than 2 are considered significant (47). STRING database (http://string-db.org) was used to build a protein-protein interaction web based on both computational predictions and experimental observations (48). All Class I and Class II phospho-proteins were imported into the database and the interaction sources selected include text mining, experiments, databases, co-expression, and co-occurrences. The minimum required interaction score was 0.700 (or high confidence). The Aspergillus genome database (AspGD) Gene Ontology (GO) Term Finder tool (http://www.aspergillusgenome.org) was used to identify enriched GO terms from imported proteins (49). Here a significance threshold of *p* value < 0.01 was used. A heat map of significantly expressed genes was created using Perseus v 1.6.5.0 (http://www.perseus-framework.org). Average Log2Fold Change is depicted, the micafungin exposure time order was preserved, and the distance metric was set to Euclidean.

##### Apparent Cell Wall Strength

To measure apparent cell wall strength in shake flask culture mycelia were removed from shake flasks (∼5 ml) during exponential growth phase and subjected to particle size analysis using a Mastersizer 3000 instrument with a Hydro SM manual sample dispersion unit following published methods (29, 50). The average mycelial size (S_90_) for each biological replicate was calculated from at least 3 timepoints (between 17 and 24 h growth) using duplicate technical replicates (*p* value set at 0.05, n≥3). In parallel with size analysis, dry cell weight measurements were taken to determine the specific growth rate of these strains.

##### Fungal Growth in Micafungin

To test whether these kinases may respond to cell wall perturbation, we grew kinase deletion mutants both in submerged culture and agar plates with micafungin. For plate growth experiments, spores were harvested from fresh fungal lawns in 10 ml of sterile diH_2_O and the spore concentration was determined. The spore solution was then diluted to 1 spore/μl and 100 μl of spore solution was spread onto both MAGV and MAGV+micafungin plates with 3 replicates. Plates were grown at 28 °C and the number of developed colonies were counted after 3 days. The critical concentration of micafungin (*i.e.* highest concentration of micafungin with no visible effect, 0.007 μg/ml) was determined by characterizing the susceptibility of the control strain, A1405 at varying concentrations of micafungin (0.001, 0.005, 0.007, 0.01, 0.015, 0.05, 0.1 μg/ml). All deletion mutants were spread on MAGV+micafungin plates (with 0.007 μg micafungin/ml) and the average number of developed colonies on MAGV+micafungin were normalized to the average number of developed colonies on MAGV plates. The significance was determined using a *t* test to compare the MAGV and MAGV+micafungin colony growth for each strain, *p* < 0.05, *n* = 3.

Next, we determined the susceptibility of fungi to micafungin during shake flask culture. Both the control strain and kinase deletion mutants were grown in YGV media to at least 1g/kg DCW (to ensure ample amount of biomass for measurements), whereupon micafungin was added (20 ng/ml micafungin per 1g/kg DCW, same as -omic experiments). Now, DCW measurements were taken over the next 6 h (usually every 1.5–2 h) to determine fungal growth rate in micafungin. Three biological replicates were used for each strain, and samples were removed after micafungin treatment for qPCR analysis, described below.

##### Quantitative RT-PCR

Samples were removed from the submerged culture with micafungin experiment at 0, 60, 90, and 120 min of micafungin exposure. Mycelia were separated from broth and frozen in liquid nitrogen and stored at −80 °C for up to 1 week. RNA extraction, purification, and cDNA conversion was completed as described in Chelius *et al.*, 2019. Primers were designed using Primer3 (supplemental File S8) (51). Three technical replicates and two biological replicates of each time point were run using a BioRad C1000 Touch Thermal Cycler with CFX96Real-Time System. The target genes were quantified with respect to the reference gene, histone (H2B), and fold change was determined with respect to the zero time point following the ΔΔCt method (52).

##### Septation Analysis by Coverslip Experiment

Coverslips with spores were prepared as described in Chelius *et al.*, 2019. After 12 h of growth in YGV media at 28 °C the first coverslip was removed from media. One drop of calcofluor white stain (18909, Sigma, Darmstadt, Germany) was added before mounting to a slide. The slide was viewed under an Olympus IX-81 inverted fluorescence microscope (Olympus, Tokyo, Japan) and 30 images were captured, for each of three biological replicates (90 images total). Each image contained at least one mycelium. Following this initial assessment, micafungin was added to media at a final concentration of 10 ng/ml. Each hour thereafter, a coverslip was removed, and images were taken until coverslips were overgrown, ∼10 h later. The images are analyzed using Image J software (53) for their projected area and the number of branches and number of septa are counted for each mycelium. The growth and branching rates are calculated as described in Chelius *et al.*, 2019 and significance was calculated based on three biological replicates (student's *t* test, *p* < 0.05).

## RESULTS

##### Dynamic Time-scale

**Fig. 1:**
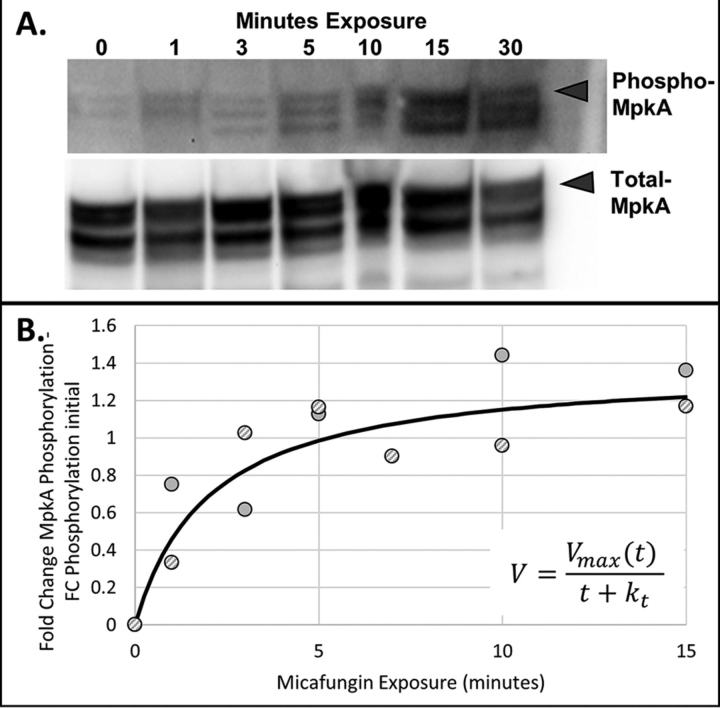
**Dynamic phosphorylation of MpkA in response to micafungin.** The control strain was grown in shake flask culture until the mid-exponential growth phase. Micafungin was added at a concentration of 20 ng/ml per 1g/kg DCW and samples (0, 1, 3, 5, 10, 15, 30 min) were removed and immediately frozen. Western blotting was used to quantify the amount of MpkA phosphorylation (*A*). Fold change band intensities from 2 biological replicates were fit to the kinetic model with *V*_max_ = 1.38 and K_t_ = 2.01 (*B*).

Eukaryotic signal transduction typically occurs over a relatively short time-scale (54). Studies in the yeast *S. cerevisiae* indicate phosphorylation events involved in signal transduction occur over a range from 5 s to 80 min, depending on the induced pathway (33, 55, 56). To determine an appropriate sampling time-scale for phosphoproteomic analysis in our study, we grew *A. nidulans* in shake-flask culture and added micafungin (β-glucan synthase inhibitor) to induce the CWIS pathway. Total and phosphorylated MpkA were determined by Western blotting (Fig. 1, supplemental Fig. S1) and modeled using a simple saturation kinetic model (*e.g.* Michaelis-Menten kinetics). The half-saturation constant, k_t_, was determined to be 2 min, meaning the majority of the dynamic MpkA phosphorylation response occurs in the first 5 min after perturbation with micafungin. We thus chose to draw samples for phosphoproteomic analysis every 30s for the first 5 min, and then at 7.5 and 10 min (13 samples total). Changes in gene expression take a significantly longer period as shown by previously published qPCR data (11) involving micafungin induced expression changes. Based on these previous findings, we drew samples for transcriptomic analysis at 0, 5, 10, 15, 20, 25, 30, 40, 50, 60, 75, 90, and 120 min (13 samples total).

##### Statistically Dynamic Phosphorylation Sites

Because of recent advancements in mass spectrometry technologies, there has been a shift from static -omic studies to dynamic studies (33, 55, 56, 57, 58, 59, 60, 61, 62, 63). However, comparison of these dynamic studies reveals significant differences in experimental design as well as data processing and analysis, such that no clear best-practice currently exists. For example, although some studies use many time points with little to no replication (33), others utilize relatively few time points, but include replicates (64). In an attempt to combine these approaches, we designed our study to include many time points (13) with multiple replicates (2 technical and 2 biological) and used label-free quantification (peak intensity) to survey phosphosite occupancy.

**Fig. 2:**
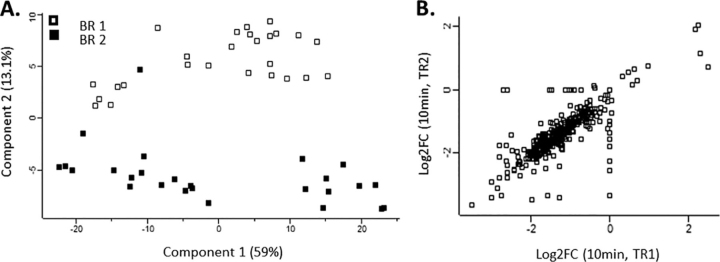
**Phosphoproteomic reproducibility.** PCA analysis of mass spectrometry data show that each biological replicate (BR1 & BR2) clusters more closely with itself than with time which can be attributed to the fast sampling time frame (0–10 min). For this reason, we applied our statistical method to each biological replicate (*A*). Quantitative technical replicate reproducibility at each time point showed tight correlation (plotted is time point 10, BR1, Pearson correlation = 0.79) (*B*).

Although our technical replicates show good correlation, there is some discrepancy between biological replicates (Fig. 2). This occurs because biological replicates are for samples taken only 30 s apart, such that even small differences in behavior shift values between time points. Previous studies have not manifested this behavior, as they have used multiple biological replicates over fewer time points, typically over much longer time scales (*e.g.* hours) (60, 61, 62).

**Fig. 3:**
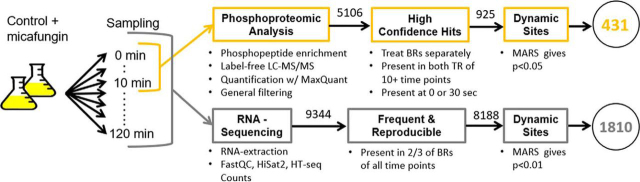
**Sampling and processing pipeline.** Fungi were exposed to micafungin and 13 samples for phosphoproteomics (0–10m) and transcriptomics (0–120m) were harvested. Three biological replicates (BRs) were used in RNA-sequencing whereas 2 were used in mass spectrometry analysis with 2 technical replicates (TRs). Overall 431 (Class I) phosphosites were found to be statistically dynamic whereas 1810 genes were dynamically expressed.

To address this issue, we developed a data-analysis pipeline to identify, with high confidence, phosposites that were significantly dynamic (*i.e.* show a significant change in occupancy after micafungin addition (Fig. 3)). We note that in the 52 different mass spectrometry runs carried out for this study, a total of 5106 phosphorylation sites were observed. However, many of these sites appeared in relatively few runs. When stringent confidence tests are applied (Materials and Methods), 1156 and 1056 phosphosites were identified in biological replicates 1 and 2, respectively, with 925 of these phosphosites (*i.e.* between 80 and 90%) in common between the two replicates (supplemental File S1).

**Fig. 4:**
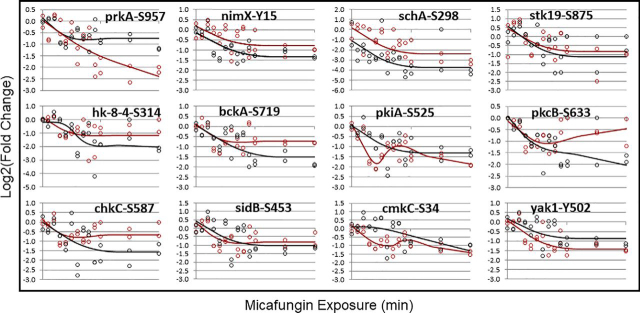
**Dynamically changing phosphosites on protein kinases for 10 min.** after micafungin exposure. Black and red circles are two different biological replicates. Lines represent MARS model fits to the data and show all phosphosites experience significant reduction in occupancy after micafungin addition.

To determine whether these 925 phosphosites were significantly changing in response to micafungin perturbation, Log2 fold change of intensity was modeled with a multivariate adaptive regression splines (MARS) model (38, 56). The quality of fit (mean square error, MSE) was used to determine if individual phosphosites were significantly changing occupancy with time (56). Fig. 4 shows MARS model fits for 12 phosphosites, all on protein kinases. Although there is some discrepancy between biological replicates, it is clear that nearly all the MARS trends (*i.e.* lines) for biological replicates are: (1) in the same direction and (2) show similar magnitude. Thus, this approach has allowed us to overcome the challenge related to comparing biological replicates taken during periods of rapid sampling.

**Fig. 5:**
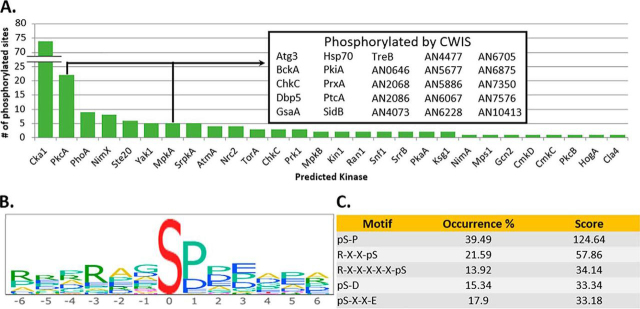
***Overall analysis of dynamic phosphorylation sites*.** NetworKIN analysis shows that a majority of the phosphorylation sites are predicted to be phosphorylated by Cka1. PkcA and MpkA (CWIS signaling kinases) phosphorylate multiple proteins showing possible new direct connections to the cell wall repair pathway (*A*). Motif-logo showing the overall enrichment of amino acids surrounding the phosphoresidue, serine, as generated by PHOSIDA (*B*). Top 5 enriched motifs identified from statistically dynamic phosphosites. The occurrence percent is the relative abundance of the motif in this data set with respect to the proteome and the score is a statistic equivalent to *p* = 10∧(-score) (C).

Using this method, we narrowed the 925 P-sites, to 431 dynamically significant phosphorylation sites in both biological replicates (Class I) and 363 in one replicate (Class II; i.e., over 700 total). From the 431 Class I sites, 51 motifs were significantly overrepresented (Fig. 5*B* and 5*C*, supplemental File S2). Further motif analysis was conducted using NetworKIN (47). Given a protein sequence, NetworKIN predicts which kinase (based on the yeast kinome) is likely to have carried out this phosphorylation. From the 431 phospho-sites, NetworKIN predicted with significant confidence that 28 different kinases phosphorylated 173 of these sites (45, 65). Of note, NetworKIN predicted that the prominent CWIS pathway kinases PkcA and MpkA phosphorylated 22 and 5 phospho-sites respectively, revealing putative, new signaling connections to the CWIS pathway (Fig. 5*A*).

##### RNA-sequencing Reveals CWIS Downstream Effectors

RNA-sequencing was carried out using shake flask cultures grown identically to the phosphoproteomic set, however the sampling time ranged from 0 to 120 min. From the 10,687 putative ORFs in *A. nidulans* (49), transcripts were identified from 9344 and of these, transcripts were present in all 13 time points and 2 of 3 replicates for 8,188 genes (supplemental File S3). From the 8188 genes, 1810 were dynamically significant (*p* < 0.01) using the MARS method (Fig. 2).

**Fig. 6:**
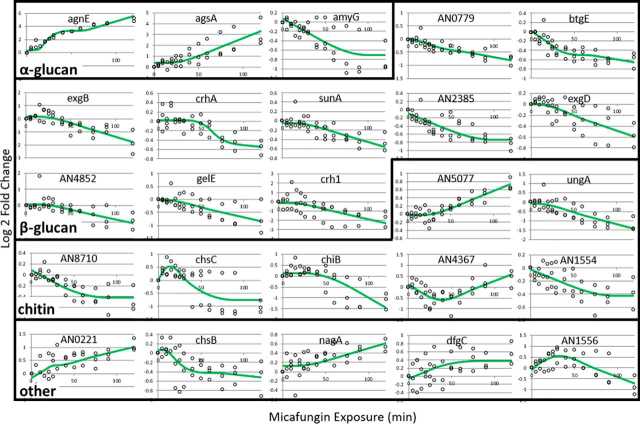
**RNA-seq data of known cell wall related genes.** The expression of 25 cell wall related genes were found to be statistically dynamic with *p* < 0.01. The green line represents is the MARS model of the three biological replicates of the log2Fold Change of gene expression (o) over 120 min of micafungin exposure.

From the statistically dynamic genes, 25 were cell wall related and 3 were probed by Fujioka *et al.*, 2007 (*agsA, chsB, chsC*) (Fig. 6; Table I) in their CWIS study. Expression profiles of these genes can be found in supplemental File S4. Of these, strict rules were used to parse out genes that were the most overexpressed (50 genes) and most under expressed (4 genes). Of the 54 most over and under expressed, 45 of these are uncharacterized genes (supplemental File S5). These uncharacterized genes may have yet unknown roles in cell wall maintenance and repair.

**Table I:** Dynamic cell wall related genes

ANID#	Gene Name	*p* value	Cell Wall Component	Up/Down	Description	Reference
AN1604	agnE	0	alpha glucan	Up	Putative 1,3-a-glucanase	deGroot et al., 2009
AN5885	agsA	0	alpha glucan	Up	Catalytic subunit of alpha-1,3 glucan synthase complex	Fujioka et al., 2007
AN3307	agsB	0.0103	alpha glucan	Down	Catalytic subunit of the major alpha-1,3 glucan synthase complex	Fujioka et al., 2007
AN3388	amyF	0.0146	alpha glucan	Up	Amylase-like family	Nakamura et al., 2006
AN3309	amyG	0.0013	alpha glucan	Down	Amylase-like family	Nakamura et al., 2006; He et al., 2014
AN1551	btgE	0	beta glucan	Down	Putative 1,3-b-transglucosylases involved in connecting the emerging 1,3-b-glucan chains to the existing b-glucan network through 1,6-b-linkages	deGroot et al., 2009
AN0933	crh1	0.0053	beta glucan	Down	Putative transglycosidases; involved in crosslinking b-glucan and chitin	deGroot et al., 2009
AN3914	crhA	0	beta glucan	Down	Putative transglycosidases; involved in crosslinking b-glucan and chitin	deGroot et al., 2009
AN4515	crhB	0.0279	beta glucan	Down	Putative transglycosidases; involved in crosslinking b-glucan and chitin	deGroot et al., 2009
AN0472	engA	0.0132	beta glucan	Down	Endo-1,3-b-glucanase	deGroot et al., 2009
AN3777	exgB	0	beta glucan	Down	Putative exo-1,3-b-glucanase	De Vries et al., 2005
AN7533	exgD	0.0013	beta glucan	Down	Putative exo-1,3-b-glucanase	De Vries et al., 2005
AN7657	gelA	0.0121	beta glucan	Down	1,3-beta-glucanosyltransferase	Fujioka et al., 2007
AN0558	gelB	0.0404	beta glucan	Down	1,3-beta-glucanosyltransferase	Fujioka et al., 2007
AN7511	gelE	0.0045	beta glucan	Down	Putative 1,3-b-transglucosylases proposed to be involved in connecting the emerging 1,3-b-glucan chains to the existing b-glucan network	deGroot et al., 2009
AN6697	sunA	0.0001	beta glucan	Down	Sun family, involved in septation, possibly b-glucosidase activity	deGroot et al., 2009
AN0726	sunB	0.0154	beta glucan	Down	Sun family, involved in septation, possibly b-glucosidase activity	deGroot et al., 2009
AN0779		0	beta glucan	Down	Putative exo-1,3-b-glucanase family	deGroot et al., 2009
AN2385		0.0002	beta glucan	Down	Mixed-linked glucanases;, hydrolyze 1,3-b-/1,4-b-glucans	deGroot et al., 2009
AN4852		0.0024	beta glucan	Down	Putative exo-1,3-b-glucanase family	deGroot et al., 2009
AN8241	chiA	0.0286	chitin	Down	Chitinase; Class III	Yamazakei et al., 2008
AN4871	chiB	0.0008	chitin	Down	Chitinase; Class V	deGroot et al., 2009
AN2523	chsB	0.0041	chitin	Down	Chitin synthase B (Chitin-UDP acetyl-glucosaminyl transferase B); Class III	Fujioka et al., 2007; Yanai et al., 1994
AN8481	dfgC	0.0372	chitin	Up	Chitinase; Class V	deGroot et al., 2009
AN1502	nagA	0.0061	chitin	Up	N-Acetyl-b-d-glucosaminidase	Kim et al., 2002
AN4234	pcmA	0.0127	chitin	Down	UDP-N-acetylglucosamine synthesis; Phosphoacetylglucosamine mutase	deGroot et al., 2009
AN9094	ungA	0	chitin	Down	UDP-N-acetylglucosamine synthesis	deGroot et al., 2009
AN0221		0.0021	chitin	Up	Chitinase; Class V	deGroot et al., 2009
AN0299		0.0218	chitin	Up	Chitinase; Class V	deGroot et al., 2009
AN0509		0.0321	chitin	Down	Chitinase; Class V	deGroot et al., 2009
AN1554		0.002	chitin	Down	Regulation of chitin synthase activity	deGroot et al., 2009
AN3122		0.0372	chitin	Down	Regulation of chitin synthase activity	deGroot et al., 2009
AN4367		0.0018	chitin	Up	Chitin synthase; Class III	deGroot et al., 2009
AN4566		0.0006	chitin	Down	Chitin synthase C (Chitin-UDP acetyl-glucosaminyl transferase C); Class I	Fujioka et al., 2007; Motoyama et al., 1994
AN5077		0	chitin	Up	Chitinase; Class V	deGroot et al., 2009
AN8710		0.0002	chitin	Down	Regulation of chitin synthase activity	deGroot et al., 2009
AN3112	ugmA	0.0172	galacto-furanose	Down	UDP-galactopyranose mutase; involved in cell wall biogenesis	Afroz et al., 2010
AN3113	ugtA	0.0111	galacto-furanose	Down	UDP-galactofuranose transporter; required for wild-type conidiophore development, conidiation, cell wall architecture, hyphal morphology and drug sensitivity; required for cell wall qalactofuranose	Afroz et al., 2010
AN0393	dfgC	0	Mannan	Down	Endo-mannanase family with a putative role in GPI-CWP incorporation	deGroot et al., 2009
AN8677	gfsA	0.0117	O-glycan	Up	Galactofuranosyltransferase involved in biosynthesis of galactofuranose antigen of cell wall O-glycan	Komachi et al., 2013
AN4390	ecmA	0.0401	unknown	Down	Putative enzyme involved in cell wall biosynthesis with unknown function; related to S. cerevisiae Ecm33	deGroot et al., 2009
AN1556	AN102	0.0015	unknown	Up	Uncharacterized	Guerriero et al., 2016

**Fig. 7:**
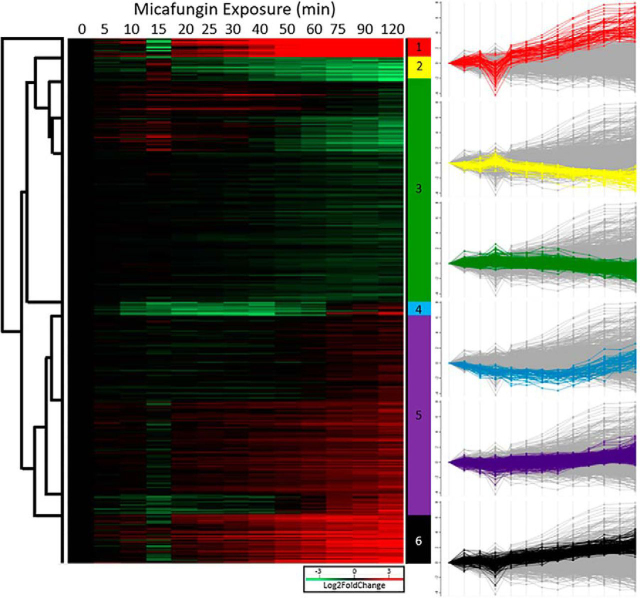
**Gene expression of statistically dynamic hits.** Here all 1810 genes are clustered in a heat map using the average Lod2FoldChange values at each time point. The profiles of 6 clusters are displayed on the right.

A heatmap of dynamically expressed genes reveals 6 gene expression clusters (Fig. 7). Gene ontology (GO) analysis of each cluster can be found in supplemental File S6. All dynamically expressed genes (1810) were searched for their GO biological process and only 805 had an associated GO term. The top 5 GO terms were transport, regulation of biological process, response to chemical, response to stress, and organelle organization (supplemental File S7). A GO term enrichment assessment revealed that the most significantly enriched group was cytoskeleton organization.

##### Cell Wall Strength of Kinase Deletion Mutants

**Fig. 8:**
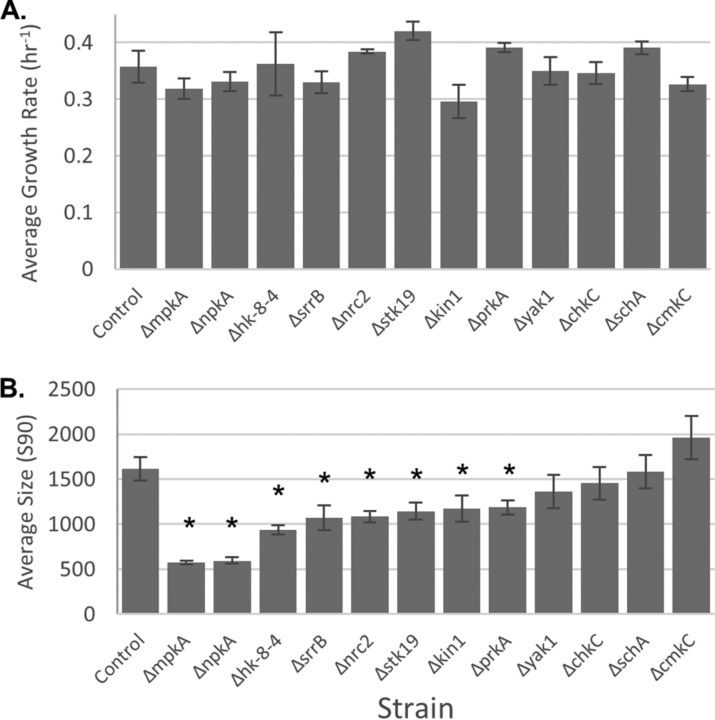
***Apparent cell wall strength of kinase deletion mutants*.** Growth rates of strains in rich medium were calculated from the exponential growth phase using dry cell weight. No significant differences between the control and mutants are observed (*A*). Particle size analysis of shake flask samples over the growth period reveal that kinase deletion mutants, Δ*mpkA*, Δ*npkA*, Δ*hk-8–4*, Δ*srrB*, Δ*nrc2*, Δ*stk19*, Δ*kin1*, Δ*prkA*, are significantly smaller than the control (*B*). A student's *t* test of mutants against the control was used with *p* < 0.05 for significance and n≥3.

In our phosphoproteomic data we observed 19 kinases and 3 phosphatases to be significantly and dynamically phosphorylated (Table II). From this group, we tested each non-essential kinase for its involvement in cell wall maintenance and repair by growing kinase deletion mutants in shake-flask culture and measuring mycelial size-distribution using laser particle-size analysis. We developed this approach previously (50) and have shown (that for similar growth rates) smaller mycelia can result from either (1) compact (*i.e.* highly branched) morphology, or (2) hyphal fragmentation during shaking. If compact morphology (deduced via microscopy) is absent, smaller mycelia are a result of hyphae breaking in the flask, suggesting fungal strains with smaller mycelia (when compared with a control strain) have weaker cell walls. We used this approach previously to show an *A. nidulans, mpkA* deletion mutant has similar growth rate and morphology to a control strain but is approximately four times smaller implying it has significantly weaker cell walls (29). We used an identical approach here, measuring growth rate and average cell size for 12 different kinase deletion mutants (Fig. 8). Compared with the control, the growth rates of all mutants were similar (*p* > 0.05). However, 8 of the 12 kinase deletion mutants had significantly smaller mycelia than the control strain (*p* < 0.05). This implies these deletion strains have weaker cell walls and are fragmenting more frequently during culture, and that the deleted kinases (PrkA, Hk-8–4, Stk19, NpkA, Nrc2, SrrB, and Kin1) are possibly involved in mediation of cell wall maintenance and repair.

**Table II:** Phosphorylated kinases and phosphatases

Gene	Residue	NetworKIN Prediction	Class	Direction	Cell Wall Strength	MF Plates	MF Flask
**Kinases**							
BckA	S 719	MpkA	I	Down	NA	NA	NA
	S 1003	MpkA	II	Down			
ChkC	S 587	MpkA	I	Down	+	+	NA
CmkC	S 34	N/A	I	Up-Down	+	+	NA
	S 475	CmkC	II	Down			
Hk-8–4	S 314	Ste20	I	Down	-	+	+
Kin1	S 257	Kin1	II	Down	-	+	NA
MkkA	S 169	PkcA	II	Down	NA	NA	NA
NimX	Y 15	N/A	I	Down	NA	NA	NA
NpkA	S 74	Snf1	II	Down	-	+	NA
Nrc2	S 433	Nrc2	II	Down	-	-	NA
PfkA	S 789	PkaB	II	Down	NA	NA	NA
	T 786	N/A	II	Down			
PkiA	S 525	PkcA	I	Down	NA	NA	NA
PkcB	S 633	PkcB	I	Down	NA	NA	NA
PrkA	S 957	N/A	I	Down	-	-	-
SchA	S 827	TorA	I	Down	+	+	NA
	S 298	PhoA	I	Down			
	S 285	SrrB	II	Down			
SidB	S 453	N/A	I	Up-Down	NA	NA	NA
	S 44	MpkA	I	Down			
	S 47	N/A	I	Up-Down			
SrrB	S 641	PkaB	II	Down	-	+	NA
SteD	S 11	Cka1	II	Down	NA	NA	NA
Stk19	S 875	N/A	I	Up-Down	-	+	-
Yak1	Y 502	N/A	I	Up-Down	+	-	NA
**Phosphatases**							
PtcA	S 41	MpkA	I	Down	NA	NA	NA
AN1358	T 390	HogA	I	Down	NA	NA	NA
PodH	S 818		I	Down	NA	NA	NA

Last three column contain phenotype data, MF is micafungin. NA: not applicable because experiment was not run on that strain (usually because kinase is essential), −: statistically significantly lower value than the control strain, +: either the same as or slightly higher than the control strain.

##### Susceptibility of Deletion Mutants to Micafungin

**Fig. 9:**
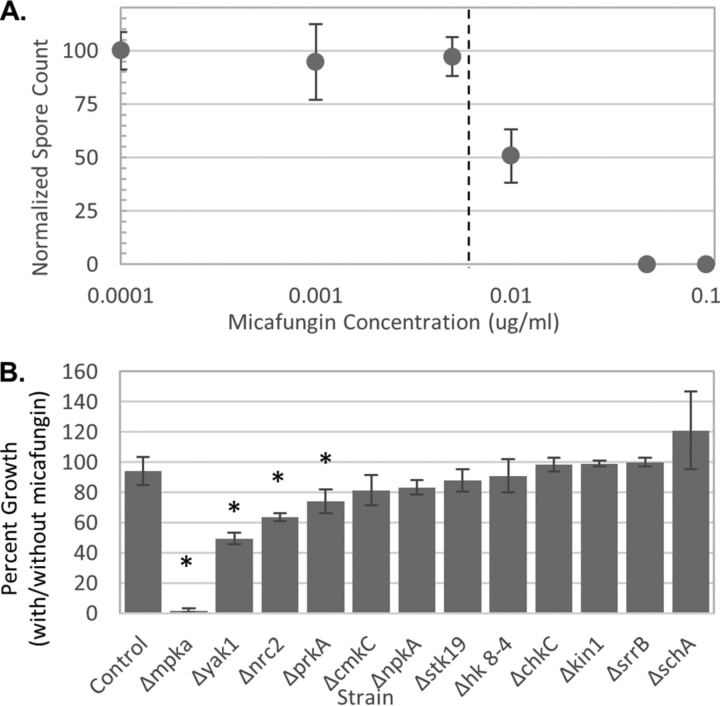
**Micafungin susceptibility of kinase deletion mutants.** 100 fresh spores of the control strain were plated on MAGV plates with various concentrations of micafungin (0–0.1 μg/ml) in order to find the minimum inhibitory concentration (0.007 μg/ml) (*A*). 100 spores of each kinase deletion mutant were grown on MAGV plates with (0.007 μg/ml) and without micafungin and the number of colonies was counted. The percent growth is displayed and Δ*mpkA*, Δ*yak1*, Δ*nrc2,* and Δ*prkA* grew significantly less than the control strain (* *p* < 0.05, *n* = 3) (*B*).

Following shake flask experiments, the same 12 kinase deletion mutants were grown on agar plates with added micafungin to test their susceptibility to a cell wall perturbant. As a member of the CWIS pathway, MpkA plays a role in regulating cell wall biosynthesis gene expression and the Δ*mpkA* strain is known to be highly susceptible to micafungin (11). To test the relative impact of micafungin on the other deletion mutants we used a highly quantifiable plate assay to determine the amount of colonial growth on micafungin agar plates. Compared with traditional spot assays, this method enables us to accurately measure small differences between strains and use statistics to confidently report susceptibility. We found that Δ*mpkA*, Δ*prkA,* Δ*yak1,* and Δ*nrc2* mutants had a significantly lower number of colonies as compared with the control (Fig. 9, Table II). Work here suggests PrkA Yak1, and Nrc2 may be involved in regulating wall-repair related genes.

##### Gene Expression of Mutants Exposed to Micafungin

**Fig. 10:**
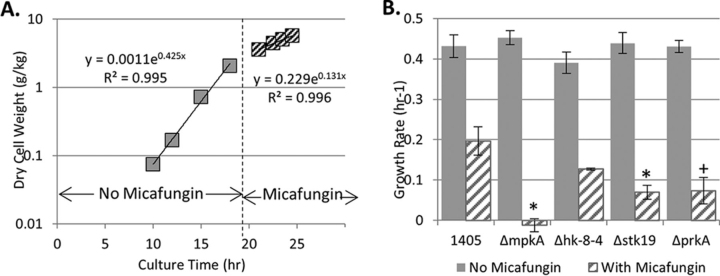
***Shake flask culture growth with and without micafungin addition*.** Four deletion mutant strains (Δ*mpkA*, Δ*hk-8–4*, Δ*stk19,* and Δ*prkA*) were grown up in YGV and after sufficient biomass was attained (1g/kg or greater), micafungin was added to culture at 20 ng/ml per 1g/kg DCW. During both growth phases (± micafungin) the growth rate was calculated using an exponential regression (*A*). Three biological replicates of each strain were grown and Δ*mpkA*, Δ*stk19*, Δ*prkA* were found to have a significantly lower growth rate when compared with the control (*p < 0.05, +*p* < 0.1). There is no difference in growth rates without micafungin (*B*).

Four kinase deletion mutants which showed reduced cell wall strength (Fig. 8; Δ*hk-8–4*, Δ*stk19*, Δ*prkA*, Δ*mpkA*) were grown in shake flasks in the presence and absence of micafungin. Micafungin was added at the same concentration as in the -omic experiments presented above. The growth rate before and after micafungin addition was measured, and Δ*mpkA*, Δ*prkA,* and Δ*stk19* all showed significantly slower growth in micafungin compared with the control (Fig. 10). For the Δ*mpkA* mutant, growth rate in micafungin appears to be negative. This is likely because of a reduction in the culture dry cell weight as this strain has relatively weak walls (29) and mycelia fragment over the extended period in the flask. The other kinases (Δ*hk-8–4*, Δ*stk19*, and Δ*prkA*) have a non-negative growth rate, but grow more slowly than the control suggesting these gene products play a role in mediating cell wall repair, but are not critical to fungal survival in this environment.

**Fig. 11:**
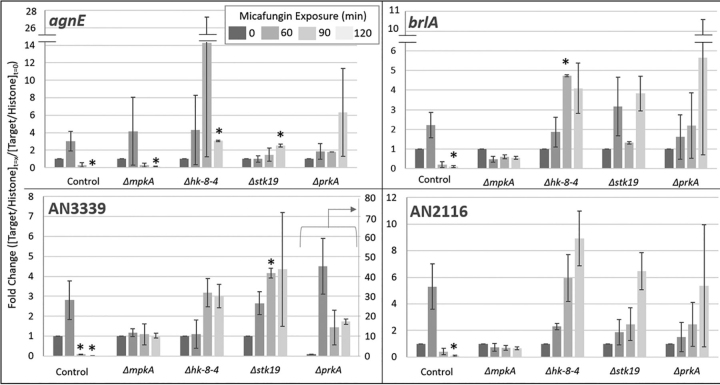
**qPCR analysis of most overexpressed genes.** The control strain and four kinase deletion mutants were grown in the presence of micafungin. Samples were harvested at 0, 60, 90, and 120 min of exposure (dark to light bars) and RNA was extracted. These samples were probed for the expression of four genes, *agnE* (alpha-1,3-glacanase) (*A*), *brlA* (a C2H2 zinc finger transcription factor) (*B*), AN3339 (uncharacterized) (*C*), and AN2116 (uncharacterized) (*D*). The target genes were normalized with the reference gene histone, *h2B*, and the fold change was determined using zero time point following the ΔΔCt method (Livak *et al.*, 2001). When compared with the control, we see that ΔmpkA does not alter its expression of brlA, AN3339, or AN2116 suggesting MpkA signaling is involved in the expression of these genes. The 3 other mutants do alter their expression, but it appears that their expression levels change around 90 min, while the control strain responds within 60 min. * indicate significant difference from the zero time point (*p* < .05, *n* = 2).

To further assess the impact of these kinases (PrkA, Stk19, and Hk-8–4) on CWIS signaling and wall repair, and at the same time explore their impact on as yet unidentified CWIS effectors, we used quantitative reverse transcription PCR (qRT-PCR). During culture of the kinase deletion mutants we measured the expression of four “fingerprint” genes (*agnE, brlA*, AN3339, and AN2116) during growth of the kinase deletions in the presence of micafungin (Fig. 11, supplemental File S8). We selected *agnE, brlA*, AN3339, and AN2116 as “fingerprint” genes as they were among the 50 most overexpressed genes in our transcriptomic data (AgnE (alpha-1,3-glucanase), BrlA (zinc finger transcription factor), AN3339 (uncharacterized), and AN2116 (uncharacterized)). Each of these genes showed a significant increase in expression during growth of the control stain in micafungin. For each gene, its expression reached a maximum at 60 min and then decreased. We note two phenomena regarding expression in the kinase mutants. First, *agnE* appears to be regulated in an MpkA-independent manner (*i.e.* Δ*mpkA* profile looks same as control). In contrast, it appears expression of *brlA*, AN3339 and AN2116 is mediated by MpkA, as they all show relatively flat profiles in the MpkA deletion mutant. Second, in the other kinase deletion strains (Δ*hk-8–4*, Δ*stk19*, and Δ*prkA*) gene expression levels take longer to reach a maximum (90–120 min). This suggests the kinases Hk-8–4, Stk19, and PrkA are involved in the temporal regulation of wall-related genes, but they are not required for their *de facto* expression.

##### Phosphorylation of GAPs, GEFs, and Transcription Factors

Phosphoproteomic analysis also shows thirteen putative transcription factors, with 16 phosphorylation sites, are significantly changing in response to micafungin addition (Table III). Those with known function in *A. nidulans* include HapC, Hsf1, RtfA, SrrA, and StuA. StuA regulates conidiophore development (66), SrrA is a stress response regulator with ties to cell wall integrity signaling (67, 68), RtfA is associated with secondary metabolism, morphological development (69), and minor effects on cell wall integrity in *A. fumigatus* (70), Hsf1 appears to regulate oxidative stress response (71), and HapC is a member of the AnCP/AnCF CCAAT-binding complex (72).

**Table III:** Phosphorylated transcription factors (TF), GAPs, and GEFs

Gene	Residue	Putative function	NetworKIN prediction	Yeast homolog	Direction
AN0463	S 1880	GEF	N/A	DCK1	Down
AN2130	S 80	GEF	N/A	CDC25	Down
AN5592	S 760	GEF	AtmA	CDC24	Down
	S 764		NimX		Down
AN5677	S 870	GAP	PkcA	RGD2	Up-Down
AN6033	S 320	GAP	N/A	GLO3	Down
AN7576	S 773	GAP	PkcA	LRG1	Down
HypB	S 1219	GEF	Ksg1	SEC7	Down
	S 781		N/A		Down
RicA	S 463	GEF	N/A	N/A	Down
	S 465		N/A		Down
AN0794	S 229	TF	Cka1	TAF9	Down
AN1500	S 427	TF	N/A	ACE2	Down
AN1944	S 6	TF	N/A	AIR2	Down
AN1984	T 3	TF	N/A	BDF1	Down
AN4694	S 316	TF	SrpkA	CTI6	Down
	S 320		Cka1		Down
AN4894	S 451	TF	Cka1	SPT7	Down
AN5898	S 255	TF	N/A	CDC36	Down
AN9358	S 2	TF	Cka1	NCB2	Down
HapC	S 18	TF	Cka1	HAP3	Up-Down
Hsf1	S 451	TF	Yak1	HSF1	Up-Down
	S 485		NimX		Down
RtfA	S 257	TF	N/A	RTF1	Up-Down
	T 259		N/A		Up-Down
SrrA	S 243	TF	N/A	SKN7	Down
StuA	S 421	TF	PkaA	SOK2	Down

From our phosphoproteomic data set we identified 11 phosho-sites belonging to 8 proteins with putative GTPase activating protein (GAP) or guanine nucleotide exchange factor (GEF) functionality. Of these, 2 GEFs, HypB and RicA, have been verified, whereas all other hits are based on homology with *S. cerevisiae* (Table III) RicA has known connections with growth and development signaling (73) whereas HypB is likely to act in COPI-mediated vesicle formation (74).

##### CWIS Role in Septation

**Fig. 12:**
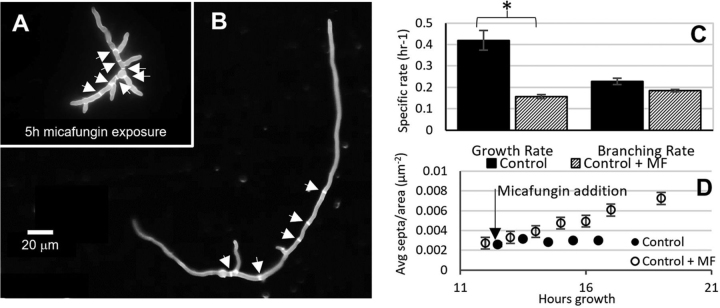
**Growth in micafungin leads to changes in septation.** Control strain was grown in rich medium for 12 h on coverslips. Micafungin was added to media (10 ng/ml) and images were taken every hour thereafter. Fungi grown in micafungin show more septa and branches (*A*) than fungi grown in rich medium (*B*). Both images were taken at 17 h total growth, white arrows point to septa. Images from this experiment were used to calculate the growth rates and branching rates for 3 biological replicates with and without micafungin (* *p* < 0.05) (*C*). When you plot septation per area, it is clear that fungi in micafungin continue to septate regardless of fungal size (*D*).

To test the role of cell wall perturbation on septation the control strain was grown on coverslips in both the presence (10 ng/ml) and absence of micafungin and stained with calcofluor white (Fig. 12) (additional images can be seen in supplemental Fig. S2). The growth rate decreases in the presence of micafungin, but the branching rate does not. This implies that branching is not a cellular process connected with cell wall stress signaling. Because septation is generally regarded as being controlled by hyphal size together with mitosis (75), when we look at the number of septa per area it is much greater for fungi grown in micafungin (Fig. 12*D*). Even though the fungi are growing slowly (compared with the control), they are undergoing septation more frequently per area. This demonstrates the role of septation in cell wall response to micafungin.

##### Phosphorylation Network

To deduce possible protein-protein interactions, we used STRING v11 to generate an interaction map (supplemental Fig. S3, Supplemental File. S9) showing connections between differentially phosphorylated proteins (48). We see three clusters form including ribosome biogenesis, calcium and cell cycle signaling, and septation and actin regulation. Of these, the septation and actin regulation cluster included several kinases (PrkA and SidB) and phosphatases (PtcA and PtcB). Proteins with altered phosphorylation related to septation initiation and formation are shown in Table IV.

**Table IV:** Phosphorylated proteins related to septation initiation and formation

Gene	Residue	Protein description	Direction
**Septation initiation signaling**			
NimX/CDC28/Cdc2	Y 15	Cyclin-dependent kinase	Down
SidB/DBF20/Sid2	S 453	Kinase of the septation initiation network (SIN)	Up-Down
	S 44		Down
	S 47		Up-Down
AspA/CDC11/Spn3	S 370	Septin	Down
**Arp2/3 Regulation**			
PrkA/PRK1/Ppk30	S 957	Serine/threonine protein kinase	Down
Pan1/PAN1/Pan1	S 1226	Predicted role in actin patch assembly	Down
AN1462/SLA1/Shd1	S 230	Predicted role in actin patch assembly	Down
AN11104/LAS17/Wsp1	T 315	Predicted role in actin nucleation	Down
	S 414		Down
AN6341/CRN1/Crn1	S 524	Predicted role in actin patch assembly	Down
	S 529		Up-Down
AN4919/ARC15/Arc5	S 139	Predicted role in Arp2/3 complex-mediated actin nucleation	Down
	S 144		Down
SlaB/SLA2/End4	S 213	Predicted actin binding protein	Down
TeaA/TEA1/Tea3	S 1379	Cell-end marker protein	Down
	S 1382		Down
AN4963/HOF1/Cdc15	S 555	Predicted Arp2/3	Down
	S 710		Down
	S 719		Down
	S 744		Down
	S 793		Down
	S 822		Down
	S 838		Down
	S 902		Down
	S 920		Down
**Actin Related Proteins**			
AN0837/NA/NA	S 663	Predicted role in actin filament severing	Down
	S 635		Down
	S 261		Up-Down
AN5677/RGD2/Rga8	S 870	Predicted GTPase activator activity	Up-Down
HypB/SEC7/Sec72	S 1219	Sec7-domain protein	Down
	S 781		Down

## DISCUSSION

**Fig. 13:**
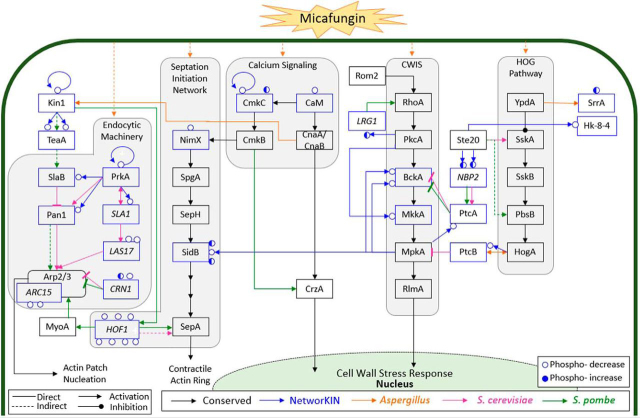
**Micafungin induced phosphorylation changes connecting conserved *A. nidulans* signaling pathways.** Dynamic phosphorylation sites are depicted as blue circles. Blue arrows show predicted phosphorylation interactions (by NetworKIN). Black arrows show the conserved signaling pathways in fungi (covered in gray boxes), orange arrows were identified in *Aspergillus*, pink arrows in *S. cerevisiae*, and green arrows in *S. pombe.* The identified phosphorylation sites combined with NetworKIN analysis suggest cell wall perturbation impacts several conserved signaling pathways in *A. nidulans*.

Our dynamic, multi-omic analysis provides insight into the signaling mechanisms and the downstream effectors involved with addressing a cell wall perturbation. Our data confidently identify statistically significant phosphorylation site occupancy changes and altered gene expression trends. We identify three main signaling pathways (septation and actin related, calcineurin and calcium signaling, and the HOG pathway) with multiple points of crosstalk between the conserved CWIS (Fig. 13) as well as other, novel protein kinases and their downstream effectors.

##### Septation and Actin-related Functions Stimulated by Cell Wall Stress

In the filamentous fungus *A. nidulans*, septum formation is an actin-dependent process that results in localized deposition of new cell wall that partitions hyphae into individual cells. Septation necessitates the formation of contractile actin rings at locations determined by the distribution of nuclei (75). Ring formation is coupled to the completion of mitosis (75, 76), and is also coordinated with growth to maintain appropriate ratios of cytoplasmic volume per nucleus (76). Our results show that septation increases in response to cell wall perturbation caused by micafungin, and our data imply potential mechanisms by which this could occur (Table IV). For example, exposure to micafungin results in reduced phosphorylation of the cyclin-dependent kinase (CDK) NimX at residue Y15, which is known to promote septum formation in *A. nidulans* (77). In addition, we also observed altered phosphorylation of the serine/threonine kinase SidB, which is a component of the septation initiation network (SIN) required for septation in *A. nidulans* (78, 79). Last, AN4693 is a homologue of *S. cerevisiae* Hof1 and *S. pombe* cdc15, both of which are key regulators of septation. Our results demonstrate that AN4693 is down-phosphorylated at multiple sites upon exposure to micafungin. In *S. pombe*, extensive dephosphorylation of Cdc15 enables interaction with multiple partner proteins that promote septum formation (80), including the SepA homologue Cdc12. Taken together, these observations suggest CWIS activation functions though the SIN and AN4693 to trigger septation. We propose that this is part of a stress response that maintains the structural integrity of damaged hyphae by confining the damage to specific regions of the mycelium.

In *A. nidulans*, polarized hyphal growth requires actin cables and actin patches; the former mediate localized exocytic delivery of vesicles at the immediate hyphal tip, whereas the latter are required for endocytosis at sub-apical sites (81, 82, 83). We identified several proteins implicated in actin patch formation that are dynamically phosphorylated because of CWIS activation (Table IV). These include regulators of the Arp2/3 complex such as PrkA, Sla1, Las17, Pan1, and Crn1. Because Arp2/3 catalyzes the formation of the branched actin filaments that form patches, these results suggest that activation of the CWIS pathway alters actin formation with subsequent effects on morphogenesis. For example, disassembly of actin patches could conceivably free up actin monomers that could be diverted toward formation of contractile actin rings to support increased septation (84). Similarly, our observation that the actin filament severing protein AN0837 is dynamically phosphorylated implies another mechanism for increasing the actin monomer pool. In addition, altered actin patch formation should impact endocytosis with resulting effects on hyphal extension. Indeed, SlaB, which localizes to actin patches and is required for endocytosis, also shows dynamic phosphorylation in response to CWIS activation.

Moreover, we observed multi-branched hyphae during micafungin exposure (Fig. 12) that most likely occurred because the extension rate of the tip slowed. The combined effect of cell wall damage and disorganization of the actin cytoskeleton may account for this. For example, the loss of actin filaments could disrupt the tip growth apparatus (85) and therefore trigger increase apical branching. We also know that a mutant (*sepA*) in which actin filament formation is severely reduced also displays apical branching (86).

A gene ontology (GO) analysis of the 1810 genes dynamically expressed in response to micafungin shows 7 of the top 10 GO terms are related to the cytoskeleton and actin organization (all categories *p* < 0.025). These include: “cytoskeleton organization,” “actin-filament based process,” “regulation of actin filament length,” and “regulation of actin polymerization,” for example (supplemental File S7). Of the 43 putative “cytoskeleton organization” genes, 18 of these have been verified (Table V). The genes *budA, fimA,* and *actA* all have implications in septum formation as its presence is transient at septation sites (82, 87). Both *ampA* and *fimA* are involved in endocytosis (82, 88). The Ras GTPase, *gapA*, has cortical localization and is believed to be involved in Ras signaling that polarizes the actin cytoskeleton (89). Microtubule (MT) dynamics and organization genes include *migA, alpA,* and *clipA* (90, 91, 92).

**Table V:** Dynamic expression of actin related genes

Gene	ANID	Description	Cluster	Avg FC	*S. cerevisiae*	Reference
actA	AN6542	Gamma-actin	Green	-	ACT1	Fidel et al., 1988
alpA	AN5521	Microtubule stabilizing, plus end-binding protein	Green	-	STU2	Enke et al., 2007
ampA	AN2516	Component of the endocytic internalization machinery	Green	+	RVS167	Araujo-Bazan et al., 2008
ampB	AN8831	Role in contractile ring assembly	Green	+	RVS161	Araujo-Bazan et al., 2008
benA	AN1182	Beta-tubulin, highly conserved component of microtubules	Green	-	TUB2	May et al., 1987
budA	AN1324	Putative actin-monomer binding protein	Green	-	BUD6	Virag and Harris, 2006
cdcA	AN5057	Putative phosphoprotein phosphatase	Green	-	CDC14	Brown et al., 2013
clipA	AN1475	Role in microtubule dynamics	Green	-	BIK1	Harris et al., 2009
fimA	AN5803	Predicted fimbrin protein	Green	+	SAC6	Upadhyay and Shaw, 2008
gapA	AN4998	Putative Ras GTPase-activating protein	Purple	-	IRA2	Harispe et al., 2008
gcpC	AN4867	Gamma-tubulin complex protein 3	Green	-	SPC98	Xiong and Oakley, 2009
gcpE	AN8120	Gamma-tubulin complex protein 5	Green	+	N/A	Xiong and Oakley, 2009
kinA	AN5343	Kinesin-family protein; involved in microtubule destabilization	Green	-	SMY1	Requena et al., 2001
migA	AN2101	Required for spindle and microtubule positioning	Green	-	KAR9	Manck et al., 2015
nce102	AN7683	Eisosomal protein; regulates sphingolipid biosynthesis	Purple	-	NCE102	Athanasopoulos et al., 2015
pphA	AN6391	Protein phosphatase	Green	-	PPH21	Son and Osmani, 2009
tubB	AN7570	Alpha-tubulin, promotes microtubule assembly	Green	-	TUB1	Kirk and Morris, 1991

##### Calcium and Calcineurin Signaling Aids CWIS Pathway

In our data, calmodulin (CaM) and its associated regulatory protein CmkC were dynamically phosphorylated in response to micafungin. CaM reversibly binds Ca^2+^ and is required for polarized growth and septation in *A. nidulans* (93, 94). Transient CaM localization occurs at sites of hyphal growth, branch emergence, and septation, which implies that it performs a critical function in localized cell wall deposition (94). CaM has also been implicated in the activation of the CDK NimX via CmkB, which is phosphorylated and activated by CmkC (Fig. 13) (95). This provides another mechanism by which CaM could impact morphogenesis in response to cell wall stress.

Key effectors of calcium signaling that regulate cell growth and cell wall integrity in *A. nidulans* are the CaM-dependent phosphatase calcineurin (CnaA) and the transcription factor CrzA (96, 97). Among proteins with roles related to calcium signaling, the genes encoding CnaA and CrzA were differentially expressed in response to micafungin (both increase expression for the first 20 min and then decrease). Accordingly, modulation of calcium signaling is likely to be an important output of CWIS activation that has broad effects on growth and morphogenesis. This is consistent with the observation that inhibition of calcineurin function exacerbates the effects of caspofungin exposure (98), and that CrzA regulates the expression of chitin synthases that are thought to reinforce damaged cell walls (99).

##### High-osmolarity Glycerol (HOG) Pathway Crosstalk

Several putative high-osmolarity glycerol (HOG) signaling proteins showed changing phosphorylation levels in response to micafungin. For example, SrrA underwent dynamic changes in phosphorylation, which is notable given that it has been previously implicated in cell wall assembly because *srrA* deletion mutants show significant resistance to micafungin (68). In addition, PtcB, which is yet uncharacterized in *A. nidulans*, was down-phosphorylated in the presence of micafungin and is a predicted target of HogA. In *A. fumigatus*, a Δ*ptcB* strain is more susceptible to cell wall perturbing agents, has increased levels of chitin and β-1.3-glucan, and has higher MpkA phosphorylation levels (100). Additional connections between components of the HOG and CWIS signaling pathways have been described in both *S. cerevisiae* and *S. pombe* (Fig. 13) (101, 102, 103, 104), thereby reinforcing the notion that pathway cross-talk might be an integral feature of the response to cell wall perturbation. In *A. nidulans*, Zhou *et al.* recently reported that hyperosmotic stress could trigger septation in a HogA- and SIN-dependent manner (105). This raises the intriguing possibility that a general function of stress-induced MAP kinase signaling in filamentous fungi might be activation of septum formation.

##### Kinases with Cell Wall Abnormalities

Eight kinase deletion strains, Δ*hk-8–4*, Δ*stk19*, Δ*prkA*, Δ*kin1*, Δ*nrc2*, Δ*npkA*, Δ*yak1*, and Δ*srrB* exhibited phenotypes consistent with cell wall integrity signaling activity (Table II). These kinases are not members of the conserved CWIS BckA-MkkA-MpkA MAPK module (11) of which only the MAPKKK, BckA (S719, S1003) and MAPKK, MkkA (S169) were identified from phosphoproteomics. Both PrkA and Kin1 have been discussed above in regards to their roles in Arp2/3 regulation. As many of these kinases have not been characterized in *A. nidulans*, here we have identified their putative function in the CWIS network for the first time by combining phenotype and phosphoproteomic data.

Hk-8–4 is a histidine kinase (HK), had weaker cell walls than the control strain (Fig. 8*B*), was phosphorylated at S314, apparently by Ste20 (based on NetworKIN analysis) which has been implicated in the SHO1 branch of the HOG pathway (106), and its deletion mutant had altered putative cell wall regulated gene expression trends from the control (Fig. 11). We hypothesize Hk-8–4 is connected with cell wall stress mediation possibly through its interaction with Ste20 and HOG. Similarly, Stk19 had decreased cell wall strength, altered gene expression (qPCR), and was sensitive to micafungin in shake flask culture. Stk19 (S875) is uncharacterized in *A. nidulans*, but has orthologues in *S. pombe* (ppk16), *S. cerevisiae* (YPL150W), and *N. crassa* (stk-19) (79) all of which have diverse cellular functions (107, 108, 109).

The *nrc2* deletion mutant was more susceptible to micafungin on solid media, had weaker cell walls, and was predicted to be auto phosphorylated at S433. Nrc2 is uncharacterized in *A. nidulans,* however its yeast homologue, Fpk1, is known to be involved in cell wall integrity signaling as it contributes (directly or indirectly) to Mpk1 (MpkA homologue) activation (110). NpkA (S74) is predicted to be phosphorylated by Snf1 and its deletion mutant (Δ*npkA*) had a weaker cell wall (this work) and showed aberrant septa formation (111).

The *yak1* mutant showed a significant decrease in colony growth in the presence of micafungin (Fig. 9*B*). We found Yak1 was dynamically phosphorylated at Y502, a tyrosine predicted to control full kinase activity (112). SrrB (S645) was predicted to be phosphorylated by PkaB (NetworKIN). Not only this, but Δ*srrB* shows sensitivity to osmotic stress (79) and had significantly smaller mycelia in shake flask culture (this work) suggesting it plays a role in CWIS (Fig. 8*B*).

##### Putative, Currently Unknown Genes Downstream of Micafungin Perturbation

Previous work identified two genes, *agsA* and *agsB,* that are downstream of the conserved MAPK signaling cascade, BckA-MkkA-MpkA (11). However, all other cell wall related genes were not dependent on this cascade. We show many interconnections between various signaling cascades which may account for this. Moreover, many genes that are not directly associated with cell wall repair are expressed at very high level in response to micafungin. Of the 50 most overexpressed genes, 45 of these are uncharacterized (Table VI; supplemental File S5). However, using computational predictions, 14 were predicted to be localized in the membrane (GO cellular component) and associated with transport functions (GO cellular function) (AN2321, AN2598, AN2781, AN2959, AN4106, AN4129, AN4173, AN7557, AN8084, AN8342, AN8595, AN8995, AN9219, and *furG*). Increased expression of membrane transport genes, in response to micafungin, may facilitate movement of cell wall material through the membrane to facilitate repair of damaged walls.

**Table VI:** 50 most overexpressed genes

Gene	Max Avg. Log2FC	Gene	Max Avg. Log2FC
AN0215	5.74	AN3339	6.87
*ivoB*	5.30	AN3996	6.15
*brlA*	4.09	AN4106	7.30
AN1261	5.90	AN4111	6.76
AN1322	4.92	AN4129	5.35
AN1600	5.37	AN4173	4.46
*agnE*	5.06	AN5546	4.79
AN1614	4.40	AN5841	5.40
AN1677	4.73	AN7557	4.51
AN1941	5.29	AN7953	5.88
AN2110	4.86	AN7954	7.57
AN2116	7.36	*furG*	5.77
AN2118	4.26	AN8084	5.71
AN2186	5.70	AN8341	4.24
AN2187	4.02	AN8342	4.12
AN2197	5.49	AN8593	6.16
AN2320	5.90	AN8594	4.23
AN2321	4.81	AN8595	4.43
AN2469	7.07	AN8597	4.08
AN2558	7.96	AN8904	4.23
AN2571	5.18	AN8995	4.69
AN2598	5.53	AN9004	5.61
AN2781	4.27	AN9191	4.87
AN2959	7.33	AN9219	4.94
AN2960	6.63	AN9490	4.09

These hits make up less than 30% of the highly expressed genes. We hypothesize, that these remaining genes are downstream effectors of the CWIS pathway and are involved in cell wall repair. AN2116 and AN3339 were probed by qPCR during micafungin exposure. They were highly expressed in the control strain after 60 min. In the Δ*mpkA* strain, the expression of these genes changed little. This suggests AN2116 and AN3339 are dependent on MpkA. AN2116 (uncharacterized) is predicted to play a role in coenzyme binding and catalytic activity (49) whereas, AN3339 (uncharacterized), is predicted to have oxidoreductase activity, transferase activity, and zinc-binding activity (49). Because of the overwhelming increase in gene expression of these 50 genes, we hypothesize they are novel downstream effectors of CWIS.

## CONCLUSION

We have applied a multi-omic methodology for identifying signaling mechanisms in response to cell stimuli. Short time-scale phosphoproteomic sampling, combined with longer-scale transcriptomic sampling, have provided a global picture of the cellular response to fungal cell-wall stress. Our statistical approach in determining dynamic effects resulted in the identification of 431 (794 with Class II) phosphorylation sites and 1810 genes. Cell wall strength and micafungin susceptibility assays provided a relatively rapid means for phenotyping relevant kinase deletion mutants and showed *hk-8–4, stk19, prkA, npkA, nrc2, srrB*, and *kin1* deletion mutants have weaker cell walls than the control. Moreover, Δ*prkA*, Δ*yak1* and Δ*nrc2* are more susceptible to micafungin on solid media, whereas Δ*prkA* and Δ*stk19* are susceptible to micafungin in submerged culture. We found many signaling molecules related to Arp2/3 regulation, septation initiation, calmodulin and calcium signaling, and high-osmolarity are changing phosphorylation and most likely activity because of micafungin perturbation. We hypothesize septation formation is being initiated via NimX, SidB, and CaM signaling, whereas PrkA and Kin1 are regulating activity of Arp2/3 and endocytosis. We propose that septation formation is a defense mechanism of fungi under cell wall perturbation to prevent further damage to internal hyphal compartments. Many uncharacterized genes were significantly overexpressed, suggesting novel, downstream CWIS effectors.

## DATA AVAILABILITY

RNA-Seq data has been deposited in the Gene Expression Omnibus (GSE136562) and mass spectrometry phosphoproteomics data in the Proteomics IDEntifications (PRIDE) repository, PXD015038 (113). MS/MS spectra can be viewed using MS-viewer (search key: yhspxctyvc; http://msviewer.ucsf.edu/prospector/cgi-bin/msform.cgi?form=msviewer).

10.13039/100000001National Science Foundation (NSF) (1517309, 1517133, 1516905) to Cynthia Chelius, Walker Huso, Samantha Reese, Alexander Doan, Stephen Lincoln, Kelsi Lawson, Raj Purohit, Ranjan Srivastava, Steven D. Harris, and Mark R. Marten
Nebraska Research Network In Functional Genomics (NE-INBRE P20GM103427–14) to Samantha Reese, and Steven D. Harris
The Molecular Biology of Neurosensory Systems CoBRE (P30GM110768) to Samantha Reese, and Steven D. Harris
The Fred & Pamela Buffett Cancer Center (P30CA036727) to Samantha Reese, and Steven D. Harris
The Center for Root and Rhizobiome Innovation (CRRI) (36–5150-2085–20) to Samantha Reese, and Steven D. Harris
