# Supplementary material for: Dynamic Transcriptomic and Phosphoproteomic Analysis During Cell Wall Stress in Aspergillus nidulans
Source: Mol Cell Proteomics. 2020 Nov 23;19(8):1310–29. doi: 10.1074/mcp.RA119.001769 (PMC8014999; doi:10.1074/mcp.RA119.001769)
Supplement: Supplementary file 1 [file mmc1.zip › 155660_2_supp_501717_q80chv.pdf]

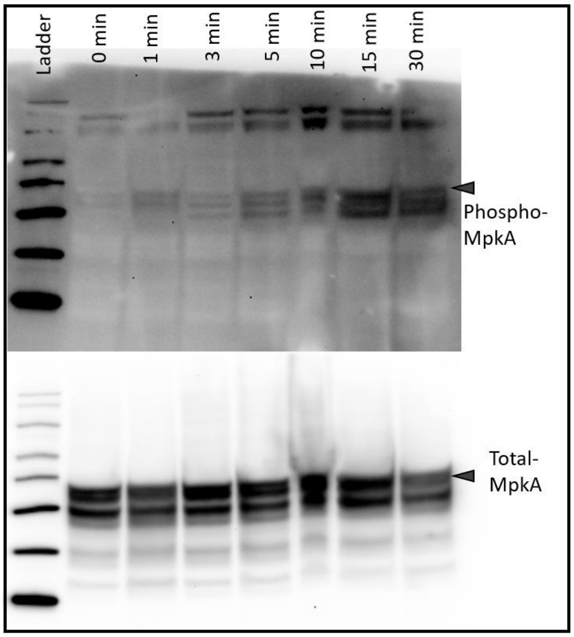

Supplemental Figure 1: *Western Blots of MpkA and phospho-MpkA.* Here full western blots are shown (same images from Figure 1).

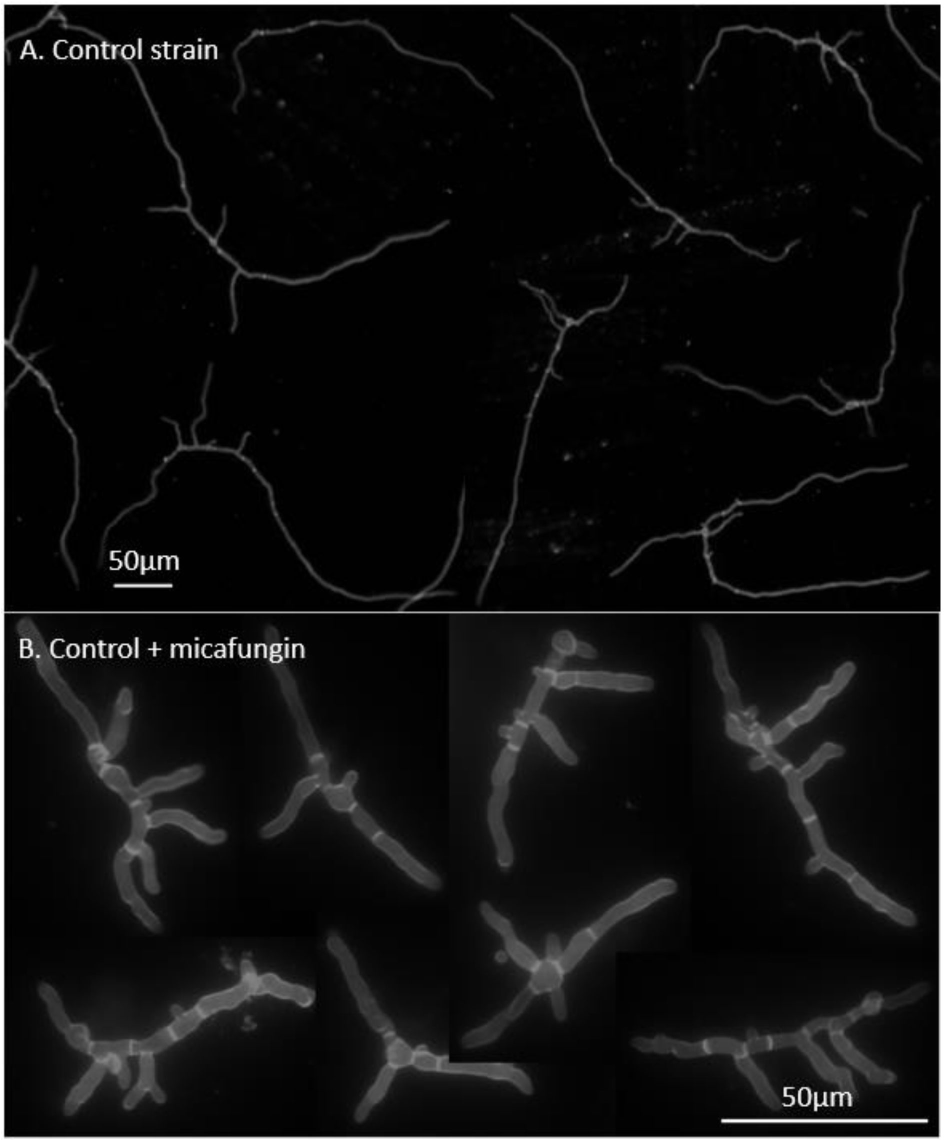

Supplemental Figure 2: *Fluorescence microscopy images of control strain with and without micafungin*. Displayed are multiple images from the coverslip experiment displayed in Figure 11. A. the control strain grown for 17 hours and stained with calcofluor white. B Control mycelia grown for 12 hours and then exposed to 10ng/mL micafungin for 5 hours and stained with calcofluor white.

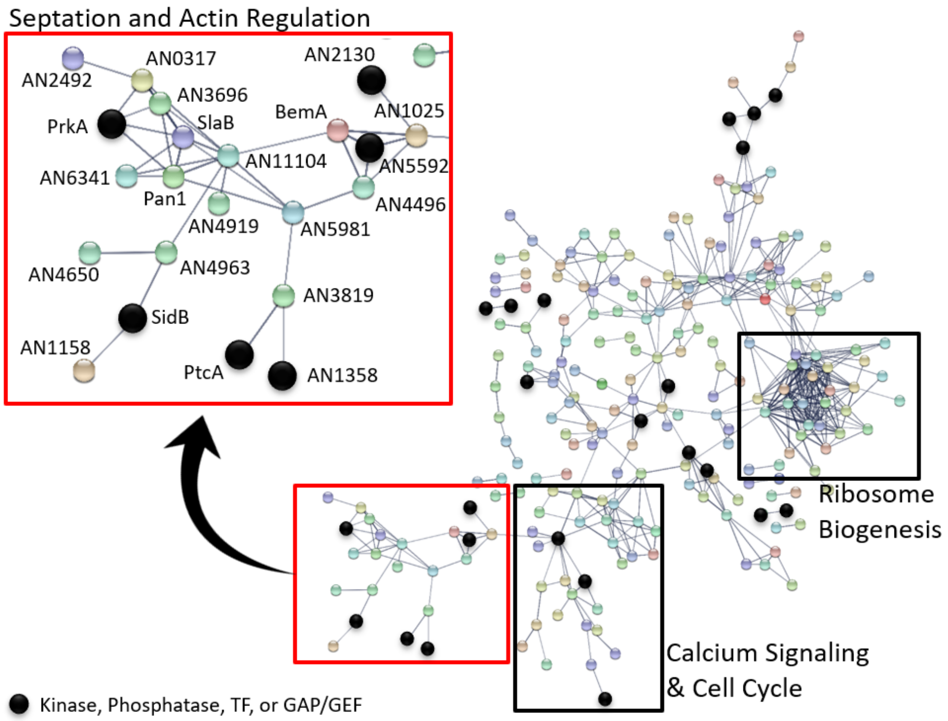

Supplemental Figure 3: *Predicted phosphorylation network of Class I and II proteins*. STRING database was used to map interactions between all Class I and II phosphorylated proteins (794). Shown here are only high confidence interactions (score>0.7). Using GO analysis on apparently clustered proteins, three main groups emerged. Black proteins represent kinases, transcription factors, phosphatases, and GAP/GEFs.
